# Supplementary material for: Metabolomics of aging in primary fibroblasts from small and large breed dogs
Source: GeroScience. 2021 Jun 16;43(4):1683–96. doi: 10.1007/s11357-021-00388-0 (PMC8492862; doi:10.1007/s11357-021-00388-0)
Supplement: Supplementary file 1 — Supplementary file1 (DOCX 485 KB) [file 11357_2021_388_MOESM1_ESM.docx]

**Supplemental Data for: Brookes & Jimenez, “Metabolomics of aging**

**in primary fibroblasts from small and large breed dogs”**

1 Table

2 Figures

| Breed | Pure **breed/Mix** | Age | Size class | Age **class** |
| --- | --- | --- | --- | --- |
| Alaskan Malamute mix | Mix | 15 y/o | Large | Old |
| Boxer | Pure breed | 13.5 y/o | Large | Old |
| Boxer/Shepherd | Mix | 13 y/o | Large | Old |
| Golden retriever | Pure breed | 11 and 13 y/o | Large | Old |
| Labrador mix | Mix | 14 y/o | Large | Old |
| Pitbull | Pure breed | 15 y/o | Large | Old |
| Australian Shepherd | Pure breed | 13 and 15.5 y/o | Large | Old |
| Rottweiler/Shepherd | Mix | 10 y/o | Large | Old |
| Siberian Husky | Pure breed | 6 and 11 y/o | Large | Old |
| Airdale terrier | Pure breed | 5 days old | Large | Young |
| Australian cattle dog | Pure breed | 3 days old | Large | Young |
| Doberman | Pure breed | 9 weeks old | Large | Young |
| German short-haired pointer | Pure breed | 2 days old | Large | Young |
| German wire-haired pointer | Pure breed | 2 days old | Large | Young |
| Labradoodle | Pure breed | 3 days old | Large | Young |
| Old English sheepdog | Pure breed | 3 days old | Large | Young |
| Rottweiler | Pure breed | 2 days old | Large | Young |
| Standard poodle | Pure breed | 4 days old | Large | Young |
| Bichon Frise | Pure breed | 12 y/o | Small | Old |
| Cocker Spaniel mix | Mix | 14 y/o | Small | Old |
| Jack Russell Terrier | Pure breed | 14 y/o | Small | Old |
| Shetland sheepdog | Pure breed | 14, 14, and 12 y/o | Small | Old |
| Shih Tzu | Pure breed | 14 y/o | Small | Old |
| Shih Tzu mix | Mix | 13 y/o | Small | Old |
| Yorkshire terrier | Pure breed | 17 y/o | Small | Old |
| Cavalier King Charles Spaniel | Pure breed | 4 days old | Small | Young |
| Corgi | Pure breed | 1 day old | Small | Young |
| Havenese | Pure breed | 4 days old | Small | Young |
| Soft coated wheaten terrier | Pure breed | 3 days old | Small | Young |
| Toy poodle | Pure breed | 3 days old | Small | Young |
| Yorkshire terrier | Pure breed | 3 days old | Small | Young |

**Table 1.** Information about breeds, ages, size and age classes, and sample sizes for dogs included in this study.

**
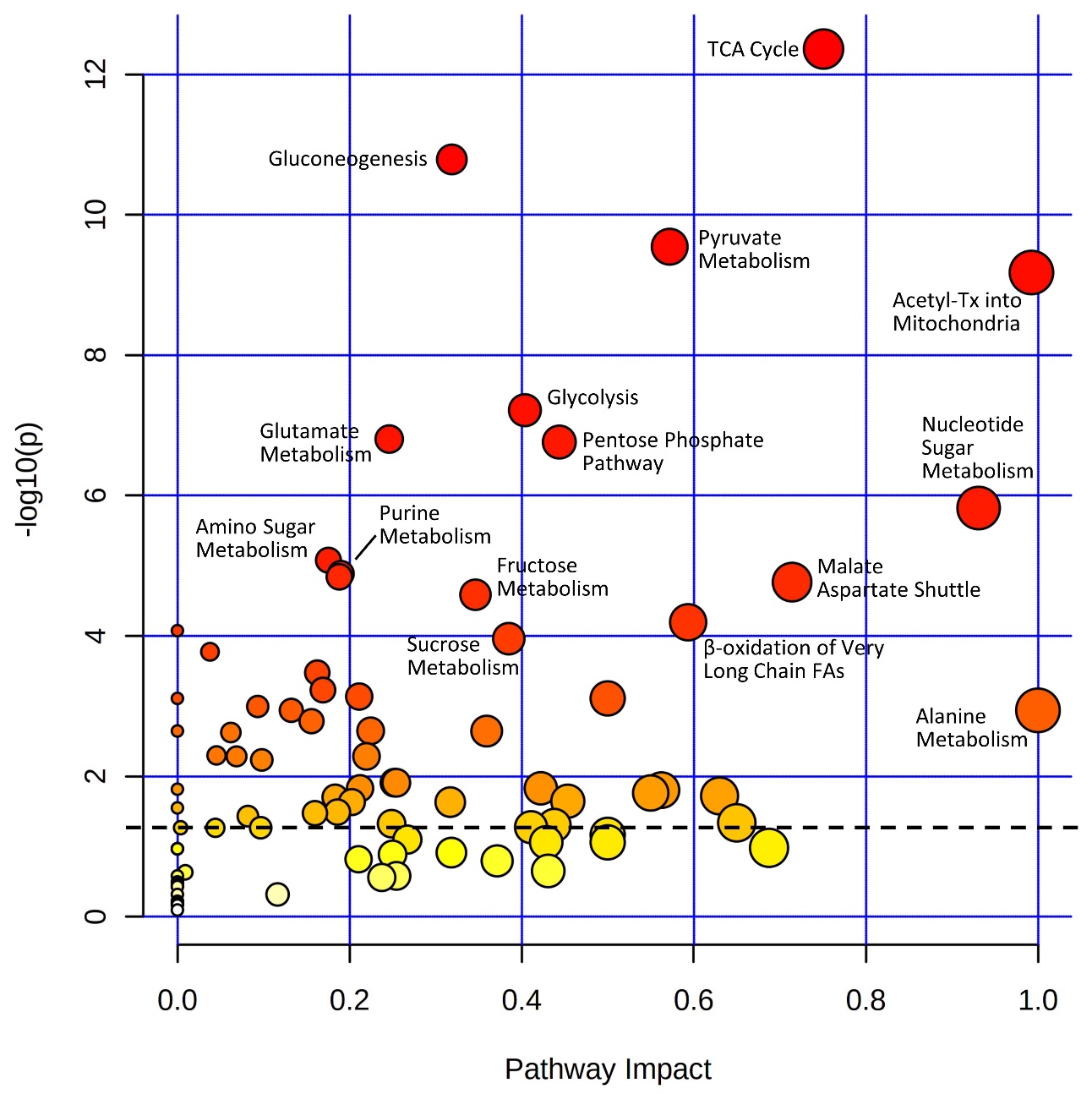
**

**Supplemental Figure 1. Pathway analysis for the metabolomic data set.**  Pathway Analysis was prepared using free web-based Metaboanalyst software. Y-axis shows -Log_10_ of p-values from the pathway enrichment analysis (Log10 of p=0.05 is 1.3, so pathways above the dashed line were considered to be significantly enriched in the analysis). X-axis shows pathway impact values from pathway topology analysis, indicating the degree of contiguous coverage within a pathway.

**
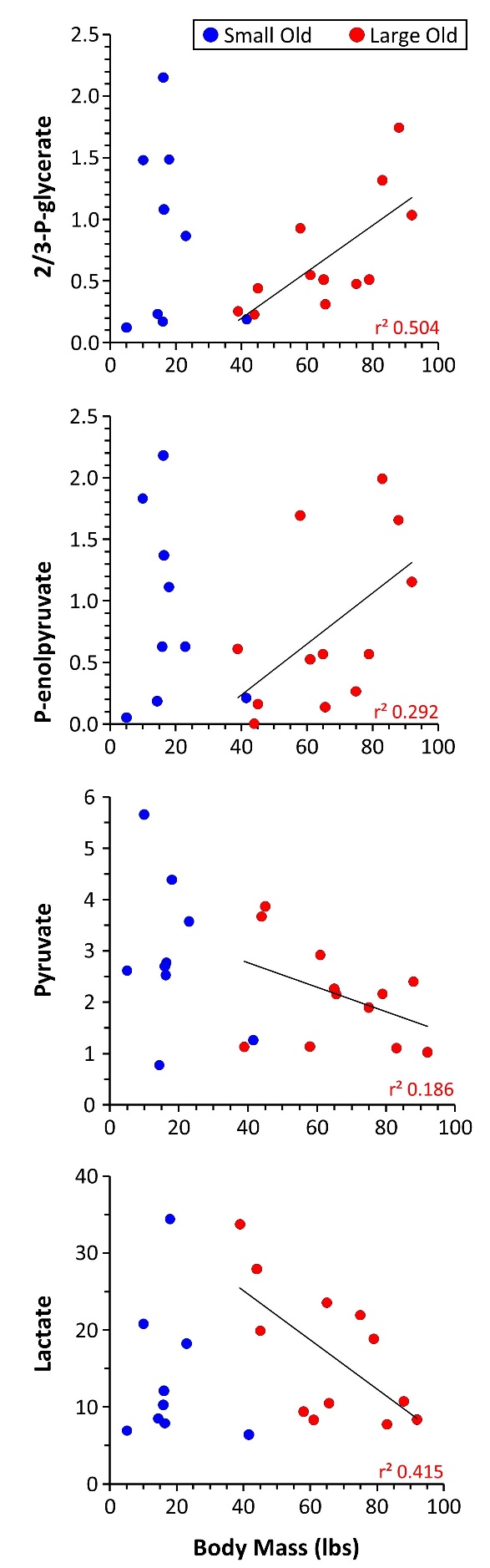
**

**Supplementary Figure 2. Correlation of metabolites with body mass in old dogs.** Correlations are shown between body mass and relative abundance (arbitrary units) of four glycolytic metabolites, in small and large old dogs. Due to insufficient variation in body mass, correlations were not calculable in young dogs of either breed size. Linear fit trendline and square of Pearson product moment correlation coefficient (r^2^) for the large old dog data set are shown on each graph.
